# Supplementary material for: Probing sustained attention and fatigue across the lifespan
Source: PLoS One. 2024 Jul 17;19(7):e0292695. doi: 10.1371/journal.pone.0292695 (PMC11253940; doi:10.1371/journal.pone.0292695)
Supplement: S1 File — (DOCX) [file pone.0292695.s001.docx]

# Supporting information

## Psychometric measures (adapted from online)

### MFI

Respondents use a scale ranging from 1 to 5 to indicate how aptly certain statements regarding fatigue represent their experiences. Several positively phrased items are reverse scored. Higher total scores correspond with more acute levels of fatigue. The scale extremes are described as “yes, that is true” and “no, that is not true”.

1. I feel fit
2. Physically, I feel only able to do a little.
3. I feel very active.
4. I feel like doing all sorts of nice things.
5. I feel tired.
6. I think I do a lot in a day.
7. When I am doing something, I can keep my thoughts on it.
8. Physically I can take on a lot.
9. I dread having to do things.
10. I think I do very little in a day.
11. I can concentrate well.
12. I am rested.
13. It takes a lot of effort to concentrate on things.
14. Physically I feel I am in a bad condition.
15. I have a lot of plans.
16. I tire easily.
17. I get little done.
18. I don’t feel like doing anything.
19. My thoughts easily wander.
20. Physically I feel I am in an excellent condition.

### VAS

You are asked to indicate on the following lines how you are feeling **right now.**

Example:

| not at all | extremely |
| --- | --- |
|  | |
| hungry  Questionnaire: | |
| not at all | extremely |
|  | |
| tired | |
| not at all | extremely |
|  | |
| sleepy | |
| not at all | extremely |
|  | |
| drowsy | |
| not at all | extremely |
|  | |
| fatigued | |
| not at all | extremely |
|  | |
| drained | |
| not at all | totally |
|  | |
| run down | |
| not at all | totally |
|  | |
| exhausted | |
| no effort at all | tremendous chore |
|  | |
| keeping my eyes open | |
| no effort at all | tremendous chore |
|  | |
| moving my body | |
| no effort at all | tremendous chore |
|  | |
| concentrating | |
| no effort at all | tremendous chore |
|  | |
| carrying on a conversation | |
| I have absolutely no | I have a tremendous |
|  | |
| desire to lie down | |
| I have absolutely no | I have a tremendous |
|  | |
| desire to close my eyes | |
