# Supplementary material for: Probing sustained attention and fatigue across the lifespan
Source: PLoS One. 2024 Jul 17;19(7):e0292695. doi: 10.1371/journal.pone.0292695 (PMC11253940; doi:10.1371/journal.pone.0292695)
Supplement: S1 Appendix — (DOCX) [file pone.0292695.s002.docx]

**S1 Appendix**

**S1 Table A. Factors affecting no-go accuracy change**

| **Effects** | **Unstandardized Coefficient B** | **Standardized Coefficient B** | **Std. Error** | **t-statistic** | **P-value** |
| --- | --- | --- | --- | --- | --- |
| Intercept | -.033 | - | .090 | -.364 | .716 |
| Age | < .001 | .086 | .001 | .860 | .391 |
| MFI mental fatigue | .007 | .143 | .006 | 1.25 | .214 |
| MFI physical fatigue | .015 | .291 | .008 | 1.72 | .088 |
| MFI general fatigue | -.015 | -.282 | .008 | -1.77 | .079 |
| MFI reduced activity | -.014 | -.258 | .008 | -1.74 | .085 |
| MFI reduced motivation | .002 | .033 | .008 | 0.243 | .809 |
| Change in Reaction Time | -.664 | -.126 | .479 | -1.39 | .169 |
| VAS fatigue change | > -.001 | -.359 | < .001 | -2.82 | .006 |
| VAS energy change | > -.001 | -.005 | < .001 | -.042 | .966 |
| VAS fatigue change VAS energy interaction | > -.001 | -.111 | < .001 | -1.16 | .250 |

MFI, Multidimensional fatigue inventory, VAS, visual analog scale

**S1 Table B. Factors affecting no-go accuracy**

| **Effects** | **Unstandardized Coefficient B** | **Standardized Coefficient B** | **Std. Error** | **t-statistic** | **P-value** |
| --- | --- | --- | --- | --- | --- |
| Intercept | .347 | - | .265 | 1.31 | .194 |
| Age | .003 | .372 | < .001 | 3.64 | <.001 |
| Total MFI | .004 | .367 | .004 | .975 | .332 |
| Pre-task VAS fatigue | < .001 | .147 | < .001 | .225 | .823 |
| Pre-task VAS energy | < .001 | .303 | < .001 | .705 | .482 |
| Total MFI Pre-task VAS fatigue interaction | > -.001 | -.374 | < .001 | -.627 | .532 |
| Total MFI Pre-task VAS energy interaction | > -.001 | -.274 | < .001 | -.798 | .426 |
| Pre-task VAS fatigue Pre-task VAS energy interaction | > -.001 | -.024 | < .001 | -.100 | .920 |

MFI, Multidimensional fatigue inventory, VAS, visual analog scale

**S1 Table C. Factors affecting go reaction time**

| **Effects** | **Unstandardized Coefficients B** | **Standardised Coefficients B** | **Std. Error** | **t-statistic** | **P-value** |
| --- | --- | --- | --- | --- | --- |
| Intercept | **5.92** | - | 0.03 | 231.48 | < .001 |
| Block | -.01 | -.385 | .001 | -8.94 | < .001 |
| Age | > -.001 | -.040 | < .001 | -.767 | .444 |
| Total MFI | > -.001 | -.021 | < .001 | -.110 | .912 |
| Pre-task VAS fatigue | < .001 | .500 | < .001 | 1.53 | .128 |
| Pre-task VAS energy | > -.001 | -.041 | < .001 | -.191 | .849 |
| Total MFI Pre-task VAS fatigue interaction | > -.001 | -.438 | < .001 | -1.47 | .142 |
| Total MFI Pre-task VAS energy interaction | < .001 | .166 | < .001 | .967 | .334 |
| Pre-task VAS fatigue Pre-task VAS energy interaction | > -.001 | -.137 | < .001 | -1.13 | .259 |

MFI, Multidimensional fatigue inventory, VAS, visual analog scale

**S1 Table D. Factors affecting pre-task state fatigue**

| **Effects** | **Unstandardized Coefficient B** | **Standardized Coefficient B** | **Std. Error** | **t-statistic** | **P-value** |
| --- | --- | --- | --- | --- | --- |
| Intercept | -321.20 | - | 71.97 | -4.46 | < .001 |
| MFI mental fatigue | 14.80 | .187 | 6.56 | 2.26 | .026 |
| MFI physical fatigue | 28.10 | .351 | 9.23 | 3.05 | .003 |
| MFI general fatigue | 60.69 | .731 | 8.98 | 6.75 | < .001 |
| MFI reduced activity | 9.84 | .116 | 8.97 | 1.10 | .275 |
| MFI reduced motivation | 7.94 | .088 | 9.00 | .882 | .380 |

MFI, Multidimensional fatigue inventory

**S1 Table E. Factors affecting pre-task state energy**

| **Effects** | **Unstandardized Coefficient B** | **Standardized Coefficient B** | **Std. Error** | **t-statistic** | **P-value** |
| --- | --- | --- | --- | --- | --- |
| Intercept | 558.45 | - | 27.65 | 20.20 | < .001 |
| MFI mental fatigue | -2.88 | -.090 | 2.52 | -1.14 | .256 |
| MFI physical fatigue | 6.75 | .211 | 3.55 | 1.90 | .060 |
| MFI general fatigue | -22.34 | -.672 | 3.45 | -6.47 | < .001 |
| MFI reduced activity | -12.60 | -.371 | 3.45 | -3.66 | < .001 |
| MFI reduced motivation | 3.48 | .096 | 3.46 | 1.01 | .317 |

MFI, Multidimensional fatigue inventory

**S1 Table F. Factors affecting state fatigue change**

| **Effects** | **Unstandardized Coefficient B** | **Standardized Coefficient B** | **Std. Error** | **t-statistic** | **P-value** |
| --- | --- | --- | --- | --- | --- |
| Intercept | 172.24 | - | 65.50 | 2.63 | .010 |
| MFI mental fatigue | -5.67 | -.109 | 5.97 | -.950 | .344 |
| MFI physical fatigue | -6.74 | -.129 | 8.40 | -.803 | .424 |
| MFI general fatigue | 5.30 | .098 | 8.18 | .648 | .518 |
| MFI reduced activity | -18.84 | -.340 | 8.16 | -2.31 | .023 |
| MFI reduced motivation | 14.78 | .250 | 8.19 | 1.80 | .074 |

MFI, Multidimensional fatigue inventory

**S1 Table G. Factors affecting state energy change**

| **Effects** | **Unstandardized Coefficient B** | **Standardized Coefficient B** | **Std. Error** | **t-statistic** | **P-value** |
| --- | --- | --- | --- | --- | --- |
| Intercept | -101.88 | - | 31.82 | -3.20 | .002 |
| MFI mental fatigue | 1.17 | .046 | 2.90 | .403 | .688 |
| MFI physical fatigue | 3.39 | .131 | 4.08 | .829 | .409 |
| MFI general fatigue | -1.35 | -.051 | 3.97 | -.341 | .734 |
| MFI reduced activity | 10.30 | .377 | 3.97 | 2.60 | .011 |
| MFI reduced motivation | -6.16 | -.212 | 3.98 | -1.55 | .124 |

MFI, Multidimensional fatigue inventory
