# Supplementary material for: Probing sustained attention and fatigue across the lifespan
Source: PLoS One. 2024 Jul 17;19(7):e0292695. doi: 10.1371/journal.pone.0292695 (PMC11253940; doi:10.1371/journal.pone.0292695)
Supplement: Attachment — Submitted filename: Response to Reviewers.docx [file pone.0292695.s003.docx]

Dear Dr Grahn,

Many thanks to you and reviewers for dedicating their time to reviewing our manuscript and offering most valuable constructive suggestions. In response to the feedback received, we have carefully revised our manuscript, incorporating all the provided comments. The following section comprehensively addresses each comment, detailing the steps we have taken to incorporate them. Changes made to the initial text are clearly highlighted in the revised manuscript. Below we provide responses to the comments and actions that we took to address them. The responses are highlighted in blue.

Yours sincerely,

**Simon Hanzal, Gemma Learmonth, Gregor Thut and Monika Harvey**

Authors of PONE-D-23-30888

Probing sustained attention and fatigue across the lifespan

**Detailed Response:**

Please include the following items when submitting your revised manuscript:

- A rebuttal letter that responds to each point raised by the academic editor and reviewer(s). You should upload this letter as a separate file labeled 'Response to Reviewers'.
  - included
- A marked-up copy of your manuscript that highlights changes made to the original version. You should upload this as a separate file labeled 'Revised Manuscript with Track Changes'.
  - included
- An unmarked version of your revised paper without tracked changes. You should upload this as a separate file labeled 'Manuscript'.
  - included

1. Please ensure that your manuscript meets PLOS ONE's style requirements, including those for file naming. The PLOS ONE style templates can be found at

[https://journals.plos.org/plosone/s/file?id=wjVg/PLOSOne_formatting_sample_main_body.pdf](https://eur03.safelinks.protection.outlook.com/?url=https%3A%2F%2Fjournals.plos.org%2Fplosone%2Fs%2Ffile%3Fid%3DwjVg%2FPLOSOne_formatting_sample_main_body.pdf&data=05%7C02%7Cs.hanzal.1%40research.gla.ac.uk%7Cccf8cd91ac1c4c7bb29208dc0c3c9b40%7C6e725c29763a4f5081f22e254f0133c8%7C1%7C0%7C638398701632430760%7CUnknown%7CTWFpbGZsb3d8eyJWIjoiMC4wLjAwMDAiLCJQIjoiV2luMzIiLCJBTiI6Ik1haWwiLCJXVCI6Mn0%3D%7C3000%7C%7C%7C&sdata=KaUC3CcYyn%2Bo6v8AUnIegjfokLdyKy%2FUd%2B4v%2FtnrRIU%3D&reserved=0) and

[https://journals.plos.org/plosone/s/file?id=ba62/PLOSOne_formatting_sample_title_authors_affiliations.pdf](https://eur03.safelinks.protection.outlook.com/?url=https%3A%2F%2Fjournals.plos.org%2Fplosone%2Fs%2Ffile%3Fid%3Dba62%2FPLOSOne_formatting_sample_title_authors_affiliations.pdf&data=05%7C02%7Cs.hanzal.1%40research.gla.ac.uk%7Cccf8cd91ac1c4c7bb29208dc0c3c9b40%7C6e725c29763a4f5081f22e254f0133c8%7C1%7C0%7C638398701632430760%7CUnknown%7CTWFpbGZsb3d8eyJWIjoiMC4wLjAwMDAiLCJQIjoiV2luMzIiLCJBTiI6Ik1haWwiLCJXVCI6Mn0%3D%7C3000%7C%7C%7C&sdata=vu5WiURtIvnyZ4qlc9KoOUgXlBSRViPEObfa8C%2FvL2Y%3D&reserved=0)

Authors’ response: We have revised the manuscript to follow the PLOS ONE template.

2. Thank you for stating the following in the Acknowledgments Section of your manuscript:

“We would like to extend our gratitude to all the reviewers and the editors who contributed to the finalisation of this article. The funders had no role in study design, data collection and analysis, decision to publish, or preparation of the manuscript.”

We note that you have provided funding information that is currently declared in your Funding Statement. However, funding information should not appear in the Acknowledgments section or other areas of your manuscript. We will only publish funding information present in the Funding Statement section of the online submission form.

Please remove any funding-related text from the manuscript and let us know how you would like to update your Funding Statement. Currently, your Funding Statement reads as follows:

“SH was supported by the Economic and Social Research Council Grant: ES/P000681/1. [https://www.ukri.org/councils/esrc/](https://eur03.safelinks.protection.outlook.com/?url=https%3A%2F%2Fwww.ukri.org%2Fcouncils%2Fesrc%2F&data=05%7C02%7Cs.hanzal.1%40research.gla.ac.uk%7Cccf8cd91ac1c4c7bb29208dc0c3c9b40%7C6e725c29763a4f5081f22e254f0133c8%7C1%7C0%7C638398701632430760%7CUnknown%7CTWFpbGZsb3d8eyJWIjoiMC4wLjAwMDAiLCJQIjoiV2luMzIiLCJBTiI6Ik1haWwiLCJXVCI6Mn0%3D%7C3000%7C%7C%7C&sdata=5Srt%2BSGrBbCw50PaSjb4hAmmJ7Vto3qbe259SPkJ4OI%3D&reserved=0)

GL was funded by the Wellcome Trust 209209/Z/17/Z.

[https://wellcome.org/](https://eur03.safelinks.protection.outlook.com/?url=https%3A%2F%2Fwellcome.org%2F&data=05%7C02%7Cs.hanzal.1%40research.gla.ac.uk%7Cccf8cd91ac1c4c7bb29208dc0c3c9b40%7C6e725c29763a4f5081f22e254f0133c8%7C1%7C0%7C638398701632587030%7CUnknown%7CTWFpbGZsb3d8eyJWIjoiMC4wLjAwMDAiLCJQIjoiV2luMzIiLCJBTiI6Ik1haWwiLCJXVCI6Mn0%3D%7C3000%7C%7C%7C&sdata=BPSdylyveWaEMk8KHxhx2sH2u3RMmOb%2F8zp1oqW5mSI%3D&reserved=0)

The funders had no role in study design, data collection and analysis, decision to publish, or preparation of the manuscript.”

Please include your amended statements within your cover letter; we will change the online submission form on your behalf.

Authors’ response: We have removed the duplicated text from the manuscript, which now only reads (page 26):

*“We would like to extend our gratitude to all the reviewers and the editors who contributed to the finalisation of this article.”*

3. Please include your full ethics statement in the ‘Methods’ section of your manuscript file. In your statement, please include the full name of the IRB or ethics committee who approved or waived your study, as well as whether or not you obtained informed written or verbal consent. If consent was waived for your study, please include this information in your statement as well.

Authors’ response: We have added the following ethics statement at the end of the participant section (page 14):

“*The study was approved by the University of Glasgow College of Science and Engineering Ethics committee (Approval number: 300200069). Digital consent was obtained from all participants by completing the online checkbox form.*"

**Reviewer 1:**

The manuscript investigated the association of fatigue (at the trait and state level) and sustained attention task performance across a sample of individuals from young to old age. Performing the attention task resulted in greater self-reported state fatigue, and state fatigue was predicted by initial pre-task trait fatigue ratings. Age was also associated with task performance measures. There were also some unexpected findings, such as a negative relationship between change in state fatigue (from before to after performing the task) and trait fatigue. The study also served as a proof of concept for the administration of the SART in an online experiment. Overall, the study is interesting and addresses an important issue – linking the subjective sense of fatigue and individual experiences with objective performance measures.

Authors’ response: We thank the reviewer for this positive assessment of our study.

My primary concerns with the paper centered on a few areas: (1) the use of a sustained attention task rather than a cognitively demanding task may induce fatigue in different ways, and produce different results – this distinction can be further clarified both in the introduction and discussion;

Authors’ response: We thank the reviewer for this valid point and detail our response further below.

(2) the use of a stepwise regression approach may produce results that are somewhat idiosyncratic, and may obscure the joint influence of multiple predictors in the models;

Authors’ response: Again, please see detailed response further below.

and (3) there are some elements of the online administration procedure that are not entirely clear, which may influence the findings, such as the monitoring of the timing of breaks for study blocks.

Authors’ response: Again, please see detailed response further below.

More specific comments related to these concerns and others are noted further below:

Introduction:

-I am a bit confused by the sentence: “We did not propose trait fatigue to affect task performance directly, but trait fatigue to affect state fatigue and thus indirectly contribute to changes in performance, and changes in state.” If certain measures of task performance are reflective of state fatigue, and trait fatigue directly influences state fatigue, then would this not be a direct affect on task performance? Perhaps this can be clarified a bit further.

Authors’ response: Thank you for pointing this out. Our main reason to believe that state fatigue would have a greater impact on performance than trait fatigue is its greater sensitivity to task-related changes. While a long-term predisposition to fatigue may be among predictors of performance, the actual state the participants are in was seen as the main indicator of performance. However, we do propose that state fatigue could be rooted in trait fatigue. We have further clarified this in the text, where it now reads (Introduction, page 6):

*“We hypothesised that trait fatigue could either negatively affect task performance directly, or that trait fatigue may predispose participants to higher levels of pre-task state fatigue. In turn, we proposed that this pre-task state fatigue may cause further in-task changes to state fatigue, and consequently to performance on the SART.”*

-the distinction and/or similarity between “fatigue” and “energy” could be further outlined.

Authors’ response: Thank you for the suggestion. Originally, we chose to use the Lee et al., (1991) VAS measure for its multi-item nature and to assess the direct momentary experience of fatigue, in preference to the single-item measure of state more typically used (Seli et al., 2013, Wylie et al., 2022). The choice of this measure with two specific subscales for “fatigue” and “energy” (and hence the choice of terminology), was thus not theory driven. Instead, wherever the original pre-registration referred to state fatigue, it denoted both subscales used in the VAS measure, the fatigue (bearing the same name) and energy subscales. However, we now distinguish between fatigue and energy in more detail in the text (Introduction, page 4):

*“There has been a tight coupling of state fatigue with energy, where several studies (13,24, 25) utilised combined measures of state fatigue comprised of energy and fatigue subscales. Although the two scales seem to be closely related, findings of divergent changes in both suggest that they constitute two related, unipolar aspects (13). Accordingly, a more in-depth investigation of state fatigue benefits from inclusion of both of the two separate subscales.”*

-Trait fatigue may also not only be defined as fatigue over a prolonged period of time, but rather it is often further characterized as an individual’s inherent tendency to experience fatigue across a range of situations. Perhaps this is not so different than the way it is defined in the text, but it may be useful to integrate this latter conceptualization of trait fatigue further into the introduction. I ask that the authors consider if this latter conceptualization provides some unique perspective to the way it is already presented in the paper.

Authors’ response: Thank you for this insightful observation. Indeed, we do not see such additional specification of trait fatigue in any way running contrary to our arguments, but as a beneficial and interesting theoretical point to elaborate. We have thus nuanced our description of MFI capturing fatigue as a trait in the introduction with the addition of the following (Introduction, page 3):

*“It has been characterised as an innate tendency to exhibit fatigue (13)”.*

And further on: *“This type of recent, self-reported fatigue has also been described as “prolonged state fatigue” (Filippi et al., 2022) in order to distinguish it from an innate tendency to become fatigued (sometimes also described as “trait fatigue”).”*

And further on in the discussion (page 25): “*as well as help to better understand innate and lasting tendency towards fatigue*.”

-Specific hypotheses associated with each aim could be added to the paper.

Authors’ response: Thank you for this consideration. We have now listed the specific hypotheses under each of the aims at the end of the introduction section (page 7), as follows:

*1) Investigate the correlational relationship between changes in state fatigue and performance changes on SART over time. Specifically, that no-go accuracy will decrease, and reaction times will increase as a) state fatigue increases, and b) state energy decreases.*

*2) Assess the relationship between no-go trial accuracy and reaction time on the SART and reported trait and state fatigue. Specifically, that no-go accuracy will be lower, and reaction times will be slower in participants with a) high trait fatigue, b) high state fatigue and c) low state energy.*

*3) Determine the relationship between subjective trait fatigue and state fatigue, as well as changes in state fatigue as a result of the task. Specifically, that trait fatigue would be a) positively correlated with pre-task state fatigue and b) negatively correlated with pre-task state energy. We also expected to observe greater SART-induced c) increase in state fatigue when pre-task trait fatigue was high, and d) decrease in state energy when pre-task trait fatigue was high.*

*4) Carry out the research project online, targeting the general population across the whole lifespan and so test the viability of an online environment for general research on sustained attention. Based on previous divergent findings, we expected to observe difference in a) no-go accuracy and b) reaction times with increasing age.*

Methods:

-was the wording adjusted for some items of the VAS-F due to regional language norms? The reliability of the scales is presumably based on the original wording used in the scales, so further comment on this adjustment is warranted.

Authors’ response: Thank you for this valid point: We have now expanded the text to point out this change and to include the effect on reliability (Materials and methods, page 9):

*“Two items of outdated language were replaced by closest possible synonyms: “worn out” to “drained”, and “bushed” to “run down” to avoid a poor understanding of the items. Subsequent internal consistency tests confirmed no impact on the measure’s reliability.”*

-Were there any other specific inclusion/exclusion criteria for participants? For example, normal vision, no history of cognitive/neurological impairment, etc. This should also be noted in the participants section of the methods.

Authors’ response: Apologies for this omission. We amended the text to read (Materials and methods, page 8):

*“Participants were admitted to the experiment if they indicated at least moderate proficiency in the English language and reported an absence of any cognitive or neurological conditions or uncorrected vision.”*

-lines 216-218: I am not following the logic of the “at chance level” phrasing here. I understand the general reasoning, which is that people could adopt a strategy where they just tend to either respond or not respond regardless of the stimulus – but I am not quite understanding how this is at chance level (like flipping a coin). These strategies would bias the response either towards higher overall accuracy (at the expense of nogo inaccuracy) or lower overall accuracy (to the benefit of nogo accuracy). Is “at chance level” the correct wording to use here?

Authors’ response: Thank you for highlighting this ambiguity. We acknowledge that the use of the word “strategy” to define the expected, deliberately erroneous, approaches that participants might take to the task was unfortunate, leading to confusion with cases where participants used valid strategies to carry out the task correctly. The text has now been amended (Materials and methods, pages 11-12):

*“As per the pre-registered protocol, participants with responses that fell into either of two anticipated deliberately erroneous approaches to completing the experiment were removed from further analysis: One was responding to all trials and withholding responses randomly at the rate of the occurrence of the no-go stimuli (> 89% go stimuli correct and < 11% no-go stimuli correct), the other to randomly respond at the rate of occurrence of the no-go stimuli (< 11% go stimuli correct and >89% no-go stimuli correct), in any of the four experimental blocks.”*

-It is unclear to me why the additional 10ms is expected for response times in the online task in comparison to in the laboratory. The expectation of increased variability seems understandable, as various systems will have different precision for detecting response speed over multiple trials. But is there a reason that a systematic delay would be expected as well, rather than just increased variability, across participants’ computers?

Authors’ response: Thank you for making this point. Our expectation was rooted in the findings of Bridges and colleagues (Bridges et al., 2020), who maintain that participants tend to access online experiments using devices with lower screen refresh rates. This can lead to a more imprecise display of stimuli in relation to participant reaction times in comparison with laboratory studies. The paper also considers lag as a factor in online experimentation. Since we have not directly compared the two, we decided to simplify the discussion to focus on variability. We have amended the text to read (Materials and methods, pages 10):

“*We expected a greater standard deviation in response times in our online sample in comparison to the conventional experiments, due to possible lower accuracy and variability of the devices the participants could use to access the task (49)*.”

-Was the number sequence randomized for each individual participant, or was the sequence of numbers the same for each person?

Authors’ response: Thank you for highlighting this matter. We have added more detail information into the method section clarifying this (Materials and methods, page 10):

*“For each participant, the numbers were sampled randomly, but with each number appearing the same number of times.”*

-It appears that a stepwise regression model was used to identify predictors that would remain in the model based on whether they reached a specific significance threshold. In general, stepwise regression can result in idiosyncratic findings that may be difficult to replicate across studies, because they increase the number of tests that are being performed and are susceptible to enhancing the impact of false positives that may be present in a given dataset. In contrast, a hypothesis driven regression model that includes a pre-specified, well-reasoned set of predictor variables is thought to produce more reliable findings. The predictors that the authors identify seem reasonable, and I suggest that the model should just include all of these predictors, especially if the authors believe that each of these predictors is a potential contributor to variance in the outcome measure. The sample size is certainly large enough for the number of predictors (I believe five in total), and the information gained from this analysis design would be more informative and potentially more reliable than a stepwise regression approach.

Authors’ response: We thank the reviewer for bringing this important point to our attention. The reviewer is correct in pointing out that a hypothesis-driven multiple linear regression model would improve the reliability of the findings. **In light of the suggestion, we have now re-run the original models and included these in the paper instead.** We have removed all references to intra- and inter-participant differences, as these could all be addressed by an inclusion of a multiple linear regression model of block averages on performance measures and block numbers as some of the predictors. Of note, rather than running full-factorial models (the main effects and all possible interactions), we have chosen to selectively include the interactions that are of theoretical interest to us. These interactions are listed in the additions to the text below, also see new appendix (S1 Appendix). After replacing the stepwise models with the multiple linear regressions, we found very similar significant effects to those reported previously, with the exception of models that examined the effect of the 5 MFI subscales on state measures and state measure changes. Originally, all trait subscales predicted pre-task state fatigue and energy. After the correction, only some of the trait subscales remained as significant predictors of pre-task state fatigue and energy. Of the two trait subscales which originally predicted the changes in state measures, only one survived this corrected approach: reduced activity. References to the original approach of hierarchical regression and examination of reaction time variability were removed from the Methods section (Materials and methods, page 12). Then, the following amendments have been made to the result section (Results, pages 16-20):

*A multiple linear regression was run to predict no-go accuracy change from each of the five individual subscales of trait fatigue, change in reaction time, change in state energy, change in state fatigue and the interaction between change in state fatigue and change in state energy. The overall model was significant F(10, 104) = 2.51 p = .010, R^2^ = .190 and only state fatigue change was found to be a predictor of accuracy change, with a negative relationship between accuracy change and state fatigue change, β < .001, t = 2.82, p = .006.*

*A multiple linear regression was run to predict correct go-trial reaction times from block number, age, total MFI score, pre-task state energy score, pre-task fatigue score and the interaction of all the subjective fatigue measures, yielding an overall significant model F(8, 451) = 11.00 p < .001, R^2^ = 0.16. Only the block number was found to be a predictor of reaction times, β = .014, t = 8.94, p < .001, with a reduction in RTs in relation to task block number, (Fig 4).*

*A multiple linear regression was run to predict no-go accuracy from block number, age, total MFI score, pre-task state energy score, pre-task fatigue score and the interaction of all the subjective fatigue measures. The overall model was significant F(8, 451) = 9.40 p < .001, R^2^ = .140. Only age was found to be a predictor of accuracy, β = .003, t = 5.89, p < .001. The older the participants were, the more accurate they were at withholding no-go responses.*

*A multiple linear regression examined the relationship between all 5 of the MFI subscales and pre-task fatigue, and another examined the relationship of the 5 MFI subscales to pre-task energy, see Fig 5.*

*The overall model predicting pre-task state fatigue was significant (F(5, 109) = 25.10 p < .001, R^2^ = .535). Pre-task state fatigue was positively related to mental fatigue (β = 14.80, t = 2.26, p = .026), physical fatigue (β = 28.10, t = 3.05, p = .003) and general fatigue (β = 60.69, t = 6.75, p < .001), but was not related to reduced activity (β = 9.84, t = 1.10, p = .275) or reduced motivation (β = 7.94, t = 0.88, p = .380).*

*The overall model predicting pre-task state energy was also significant (F(5, 109) = 29.11 p < .001, R^2^ = .572). Pre-task state energy was negatively related to general fatigue (β = 22.34, t = 6.47, p < .001) and reduced activity (β = 12.60, t = 3.66, p < .001), whilst showing no relationship to mental fatigue (β = 2.88, t = 1.14, p = .256), physical fatigue (β = 6.75, t = 1.90, p = .060) or reduced motivation (β = 3.48, t = 1.01, p = .317).*

*However, state change only modestly corresponded to the MFI scores. The overall model was significant (F(5, 109) = 2.38 p = .043, R^2^ = .099). State fatigue change was negatively related to reduced activity (β = 18.84, t = 2.31, p = .023), with a small effect size. This meant that participants who had higher reduced activity scores had a smaller increase in their state fatigue during the task. There were no other relationships between state fatigue change and any of the other 4 MFI subscales: mental fatigue (β = 5.67, t = .950, p = .344), physical fatigue (β = 6.74, t = .803, p = .424), general fatigue (β = 5.30, t = .648, p = .518) or reduced motivation (β = 14.78, t = 1.80, p = .074). The same model was run to predict state energy change from the five MFI subscales (F(5, 109) = 3.10 p = .012, R^2^ = .125). Again, reduced activity was also a positive predictor of energy change, β = 10.30, t = 2.60, p = 0.01 with a small effect size. This showed that participants with higher reduced activity scores reported less energy loss during the task. No relationship was found with the other scales: mental fatigue (β = 1.17, t = .403, p = .688), physical fatigue (β = 3.38, t = .829, p = .409), general fatigue (β = 1.35, t = .341, p = .734) and reduced motivation (β = 6.16, t = 1.55, p = .124). Therefore, the direction of the relationship was the opposite of our pre-registered prediction (we predicted that we would observe more task-induced fatigue and energy loss in participants who started the experiment with high levels of reduced activity) (see Fig 6).*

Further, in line with these revisions to the statistical models, we have also amended the limitations section (Discussion, pages 23-24):

*“The original pre-registered models were based on concrete linear predictions between one predicted and one predictor variable, but we considered several variables within one model, not all of which were directly mentioned in the pre-registration. Nonetheless, the models broadly reflect the anticipated pre-registered relationships. This approach helped us to detect the unanticipated link between increased trait fatigue and reduced state fatigue change. However, this remains an initial exploratory finding.”*

-The effects of fatigue on the SART are being estimated by comparing performance between the first and fourth blocks. However, on the fourth block, participants are still starting the block after having a break. So it is somewhat unclear how much performance should be expected to decline if there are sufficient breaks between each block.

Authors’ response: We agree with the reviewer that it is possible for individually paced breaks, as opposed to fixed-length breaks, to impact changes in experimental measures (Helton & Russel, 2015). The matter requires further research, with some findings indicating that even at 10 minutes, breaks do not fully reset responses in subsequent blocks of attentional tests (Cassarino et al., 2019). The nature of the online experimentation does not allow for as strict an experimental control as laboratory studies, so it is important to acknowledge the potential differences between outcomes in online and in-person experimentation (but note that we still found a decrement). We have now expanded the discussion (Discussion, page 23):

*“They also demonstrate the feasibility of obtaining decrement effects despite reduced experimental control over participants than in laboratory settings, such as less influence on the lengths of breaks between blocks.”*

Furthermore along these lines: was there any data collected regarding the length of the breaks that participants took between blocks?

Authors’ response: Our survey collection platform (Qualtrics) did not allow us to accurately measure the time spent on the breaks between experimental blocks. However, to address concerns about the impact of break length, we investigated all the information we had available, which was the total survey duration time. Here, we observed no relationship between fatigue change scores and survey duration (F(1, 113) = 3.60, p = .060) and neither was there any relationship between energy change scores and survey duration (F(1, 113) = .01, p = .923), indicating that longer breaks, as part of generally more time spent on the whole experiment, did not significantly reduce the induction of fatigue. Even after a removal of outliers (participants with a duration 2 SD over the mean (n = 5)), there was no relationship found between task duration and either fatigue (F(1, 108) = .430, *p* = .516) or energy (F(1, 108) = .01, *p* = .949).

Did participants take different lengths of breaks, on average, based on the age stratification? This is a potential confound in the data that should be reported, and which could also potentially be further controlled for in the statistical analyses.

Authors’ response: There were no traceable age differences in task duration to suggest that older people took longer breaks than younger people, F(1, 113) = 4.00, p = .145.

Results:

-What was the reason for log-transforming the reaction time data? Were the reaction time data skewed? If they were normally distributed, then why is the log transformation necessary?

Authors’ response: We agree with the reviewer that this reason should be outlined in greater detail. The reaction times were log-transformed as there was a combination of a positive skew and a lack of a floor effect, both indicating suitability of the data for log transformation. More information on the log transformation was added (Results, page 15-16):

*“After removal of the trials with very short reaction times (< 150ms, 3.0% of data), and although the sample mean reaction times matched the times expected based on prior studies (364.47ms, SD = 5.15ms), they appeared not to be normally distributed (they had a heavy rightward skewness (2.02) and were leptokurtic (12.44)). So, the reaction times were corrected by log-transformation, leading to more acceptable skewness (0.50) and kurtosis (4.54). The mean within-participant standard deviations in reaction times were 99.87ms (SD = 7.76) and normally distributed (skewness = 0.22, kurtosis = 2.69).”*

-A table should be included that provides a full overview of the relevant statistics for the regression models.

Authors’ response: Thank you for the suggestion. We have now included all of the regression model tables in Supporting Information (S1 Appendix) and added the reference to the text (Results, page 16):

*“The full regression table is available in the Supporting Information (Table in S1 Appendix).”*

-The left panel in figure 4 appears to show identical mean reaction time and SD of RT across the four blocks of the task. However, the trendline in the right panel, and the analyses reported in the text, both appear to indicate that there is a reduction of RT overall across the blocks. It seems that there is a discrepancy between these figures, or is there some other additional information I am missing here? A similar question applies regarding the variability (SD) around the mean.

Authors’ response: We agree with the reviewer that the left panel of Figure 4, while showing the exact range of all the reaction times, was uninformative for block-level comparisons. Consequently, the figure was fully revised. It now shows participant and block average reaction times as opposed to trial-level data and displays standard deviation bars. The original plots included standard error bars which did not facilitate comparison between blocks. Please see the revised Figure 4. Furthermore, the figure legend was corrected to reflect this change (Results, page 17):

*“Distribution of reaction times at trial block level across the four blocks including mean reaction times and standard deviations with reaction times within each participant across the four task blocks distinguished by colour (including the overall linear trend across the four blocks).”*

-Were corrections for multiple comparisons applied to other analyses that did not include age and gender? If that is not the case, then is unclear why the correction was applied only when these factors were present in the analyses.

Authors’ response: Thank you for this observation. The matter is related to the previously addressed point regarding stepwise regressions. Since these statistical comparisons have now been merged into larger models, the problem of multiple comparisons is now redundant. We have now removed references to it in Method, pages 12-13, and Results, page 20.

Discussion:

-I am curious if the improving performance on the SART as a function of increasing age is influenced in some way by the level of fatigue an individual experiences (either trait or state). Do individuals with greater reported fatigue not show the same level of improvement across task blocks? The discussion on page 25 suggests that the older participants may have been more interested in the task – but I wonder if this interest would wane for individuals that are highly fatigued, and if this effect would be stronger in older compared to younger participants.

Authors’ response: Thank you for raising this interesting and insightful point. We agree that underlying motivational factors may contribute to the potential advantage that older adults appear to have in overcoming fatigue and task difficulty (Carr et al., 2022). While there is a degree of saturation in younger cohorts, experimental participation has been reported to be more meaningful to older adults (Ryan & Campbell, 2021). Participation in research may thus activate a reward mechanism that operates more effectively in older compared to younger cohorts, allowing the older participants to maintain (or even improve in) some aspects of their performance during the task compared to younger adults (Carr et al., 2022). At the same time, the present findings did not explicitly aim to explore differences between the age groups in terms of their motivation towards participation. Overall, this topic is of considerable interest to us and merits subsequent research to be carried out. We have therefore amended the future directions section to outline a call for further research on this age difference (Discussion, page 25):

*“In contrast, older adults may experience a more innate motivational drive to excel at the task (77,78). Future work could investigate this phenomenon in more detail by utilising motivational manipulations and probing attitudes to experimentation in participants.”*

-The ability to sustain attention (as represented in the SART) could be conceptualized as overlapping with, but also distinct from, fatigue in different ways. Cognitive fatigue, in particular, is thought to be induced when people have to engage in a cognitively challenging task with high demands. In contrast, the SART is not necessarily cognitively challenging to the same degree as some other tasks. So the findings of the study could be different based on whether one is measuring sustained attention vs. a cognitively demanding task, such as for example a challenging dual-task paradigm or task with high working memory or executive demand. This nuance of the findings could be further outlined in the discussion (and maybe it would be worth addressing this further in the introduction as well).

Authors’ response: Thank you for raising this key point. We acknowledge that the SART may not be a highly cognitively challenging task for many individuals, but it does require prolonged periods of sustained attention and concentration in order to achieve a low error rate. We completely agree that a sustained attention task may differ from other challenging tasks in their demands, and therefore could potentially induce different types, or levels, of fatigue. We have expanded on this issue in both the introduction and the discussion section as follows:

(introduction, page 3) *“One frequently adapted model describes fatigue as a change from baseline state in response to either physically or mentally challenging tasks, which induces a depletion of cognitive resources and lowered vigilance (6). Another mechanism of fatigue induction could be via boredom, where repetitive and unstimulating tasks lead to an inability to maintain sustained attention (7).”*

(discussion, pages 21-22) *“Our selection of the SART to induce fatigue was motivated by its ability to induce errors in the withholding of a behavioural response caused by lapses in sustained attention. The prolonged, and repetitive nature of this task may have induced a different type (or a different degree) of fatigue compared to a task that, for example, depletes cognitive resources due to high complexity (7, 70) or involves high working memory load (71). It may be the case that participants with high trait fatigue did not experience greater state fatigue changes because they had a higher tolerance for unstimulating, repetitive tasks, and it is possible that the use of a challenging task may have produced different results in this regard, which should be investigated in future research.*

Other minor comments:

Abstract: perhaps some additional text could be added to provide further context for the meaning of “reduced energy” in the abstract.

Authors’ response: The text was corrected to (Abstract, page 2): *“And an accompanying reduction in subjective energy rating.”*

-line 38, typo? – “the3”

Authors’ response: The text was corrected (page 3).

-line 56: MFI should be defined at first use.

Authors’ response: The text has been corrected (page 3).

-line 89: check phrasing of the sentence.

Authors’ response: The text has been corrected (page 4).

-line 124: should just say “SART” without “task” afterwards, I believe, as the “task” is redundant with the “T” in the acronym.

Authors’ response: The text has been corrected throughout.

-I am unclear what is meant by the phrase “a balanced mean” on page 16.

Authors’ response: The text has been corrected (page 15): *“The sample had a mean score around the midpoint of the scale.”*

Later on, the text now reads *“The results were similar to those found in other populational studies.”*

-line 476, typo: “reaction times”.

Authors’ response: The text has been corrected (page 22).

-line 480, phrasing: “the laboratory setting”

Authors’ response: The text has been corrected (page 23).

**Reviewer #2:** Overall I think this study is a very good study

Authors’ response: Thank you very much for this positive assessment of our study.

however, this study ignores much of the published works of O'Connor, Boolani and Filippi. The work of Fillipi (Fillipi, et al, 2022) suggests that what you are measuring with MFI may be prolonged state fatigue and not necessarily trait fatigue. Further, works by both Boolani and O'Connor suggest that trait fatigue is the long-standing pre-disposition to fatigue and not how someone has felt lately, as the MFI instructions suggest.

Authors’ response: Thank you for this valuable point which was also made by reviewer 1. We have now incorporated a discussion of this more nuanced understanding of fatigue on page 4:

*“There has been a tight coupling of state fatigue with energy, where several studies (13,24,25) utilised combined measures of state fatigue comprised of energy and fatigue subscales. However, while the two scales seem to be closely related, findings of divergent changes in both suggest that they constitute two related, unipolar aspects (13). Accordingly, a more in-depth investigation of state fatigue benefits from inclusion of both of the two separate subscales.”*

And elsewhere in the text:

*“It has been characterised as an innate tendency to exhibit fatigue (13).”*

Further, recent work by Boolani (>5 manuscripts) support that trait mental and physical energy and fatigue modify state energy and fatigue in the direction that your results suggest (high trait fatigue results in a lower increase in state fatigue than low trait). This may be due to the fact that those who normally feel fatigued (high trait fatigue) do not have much reserve left and are already starting more fatigued than their counterparts so they can't possibly feel worse. Boolani and colleagues have noted these changes in multiple studies. Their control interventions may provide you with comparisons.

Authors’ response: Thank you for this excellent point. It is very encouraging to see this explanation for some of our findings which ran contrary to our expectations. We now include references to this work in our discussion (Discussion, page 22):

*“The findings parallel those of Boolani and colleagues (72), who likewise detected that higher trait fatigue in individuals meant the inability to get further fatigued. The effect has further been suggested to occur due to an already depleted cognitive reserve on part of the participants, preventing any further rises in fatigue (73).”*

Another issue with this work is that the authors do not acknowledge that energy and fatigue are two distinct uniolar moods with their own distinct biological, behavioral and biomechanical correlates.

Authors’ response: We agree with the reviewer and have now addressed the distinction of the two concepts (as described in response to reviewer 1 (page 4).

Secondly, I downloaded your data and did some visualizations and ran Shapiro-Wilks tests for normality and your pre and post VAS scales were not normally distributed (after calculating for fatigue and energy scores). I ran the same for deltas and saw that while fatigue was normally distributed, energy was not. Did you apply any sort of data transformation techniques to these scores?

Authors’ response: We thank the reviewer for raising this concern regarding the normally distributed scores. We re-inspected the distribution of the variables and pre-task state fatigue, in particular, seems to show a combination of a floor effect with a slight positive skew. We believe that this floor effect is interesting in its own right and likely reflects a portion of the population who experience minimum fatigue when participating in online experiments. It is reasonable to believe that some people experience no fatigue at all before the start of the task. Deltas show the reverse pattern, where fatigue change follows a normal distribution, and energy does not.

We further note that correction is not customary for state psychometric measures (Shigihara et al., 2013, Trecroci et al., 2020, Wylie et a., 2022). So, in contrast to reaction times where log-transformation was applied, the adjustment of the fatigue and energy measures may be less optimal in this case, as it may mis-represent the state of the sample prior to the start of the task.

We would thus like to argue that an inclusion of the non-corrected measures provides a more accurate representation of the fatigue states participants experience at the beginning of participation in an online experiment. Furthermore, adjustment of the psychometric measures may implicate their relationship to other non-corrected measures and blur the trait- and state- links. So, we decided to instead report our findings as potentially characteristic of the state of the participants in an online experiment (Discussion, page 23):

*“It also provides a characteristic profile of fatigue and baseline fatigue rates prior to experimentation in online samples across the whole adult lifespan, with the exception of very advanced age, as well as the influence of fatigue levels over the course of the experiment.”*

Same with age, you had more people in the late teens/early 20s in the 18-29 cohort, the same goes for the 70-81.

Authors’ response: Despite Figure 1 showing an apparently balanced age distribution until the age of 75, we followed the reviewer’s advice and took a closer look to investigate the concern of imbalanced age groups. In the sample 18-81, there were an average of 1.80 people per year of life, with a SD of 1.44 and a range from 0-6. Therefore, whilst there was a very slight over-representation of ages at the beginning of some recruitment cohorts, arguably the age data is balanced. However, a concluding remark in the limitations on generalisation regarding the oldest age bin was added to account for this slanted end of the age range as follows (Discussion, page 23):

*“It also provides a characteristic profile of fatigue and baseline fatigue rates prior to experimentation in online samples across the whole adult lifespan, with the exception of advanced age, as well as the influence of fatigue levels on the course of the experiment.”*

I think the statistics need a closer look as distribution of data may impact your models.

Authors’ response: We thank the reviewer for investigating the variables and point to our revised statistical approach of using multiple linear regression, also in light of reviewer 1’s comments.

References:

Bridges, D., Pitiot, A., MacAskill, M. R., & Peirce, J. W. (2020). The timing mega-study: comparing a range of experiment generators, both lab-based and online. *PeerJ*, 8, e9414. https://doi.org/10.7717/peerj.9414

Carr, D. C., Tian, S., He, Z., Chakraborty, S., Dieciuc, M., Gray, N., Agharazidermani, M., Lustria, M. L. A., Dilanchian, A., Zhang, S., Charness, N., Terracciano, A., & Boot, W. R. (2022). Motivation to engage in aging research: Are there typologies and predictors? *The Gerontologist*, 62(10), 1466-1476. https://doi.org/10.1093/geront/gnac035

Cassarino, M., Maisto, M., Esposito, Y., Guerrero, D., Chan, J. S., & Setti, A. (2019). Testing attention restoration in a virtual reality driving simulator. *Frontiers in Psychology*, 10, 250-250. https://doi.org/10.3389/fpsyg.2019.00250

Filippi, M., Krähenmann, R., & Fissler, P. (2022). The link between energy-related sensations and metabolism: Implications for treating fatigue. *Frontiers in Psychology*, 13, 920556-920556. https://doi.org/10.3389/fpsyg.2022.920556

Helton, W. S., & Russell, P. N. (2015). Rest is best: The role of rest and task interruptions on vigilance. *Cognition*, 134, 165-173. https://doi.org/10.1016/j.cognition.2014.10.001

Lee, K. A., Hicks, G., & Nino-Murcia, G. (1991). Validity and reliability of a scale to assess fatigue. *Psychiatry Research*, 36(3), 291. https://doi.org/10.1016/0165-1781(91)90027-M

Ryan, A. D., & Campbell, K. L. (2021). The ironic effect of older adults’ increased task motivation: Implications for neurocognitive aging. *Psychonomic Bulletin & Review*, 28(6), 1743-1754. https://doi.org/10.3758/s13423-021-01963-4

Seli, P., Carriere, J. S. A., Levene, M., & Smilek, D. (2013). How few and far between? examining the effects of probe rate on self-reported mind wandering. *Frontiers in Psychology*, 4, 430-430. https://doi.org/10.3389/fpsyg.2013.00430

Shigihara, Y., Tanaka, M., Ishii, A., Kanai, E., Funakura, M., & Watanabe, Y. (2013). Two types of mental fatigue affect spontaneous oscillatory brain activities in different ways. *Behavioral and Brain Functions*, 9(1), 2-2. https://doi.org/10.1186/1744-9081-9-2

Trecroci, A., Boccolini, G., Duca, M., Formenti, D., & Alberti, G. (2020). Mental fatigue impairs physical activity, technical and decision-making performance during small-sided games. *PloS One*, 15(9), e0238461-e0238461. https://doi.org/10.1371/journal.pone.0238461

Wylie, G. R., Pra Sisto, A. J., Genova, H. M., & DeLuca, J. (2022). Fatigue across the lifespan in men and women: State vs. trait. *Frontiers in Human Neuroscience*, 16, 790006-790006. https://doi.org/10.3389/fnhum.2022.790006
