# Supplementary material for: Probing sustained attention and fatigue across the lifespan
Source: PLoS One. 2024 Jul 17;19(7):e0292695. doi: 10.1371/journal.pone.0292695 (PMC11253940; doi:10.1371/journal.pone.0292695)
Supplement: Attachment — Submitted filename: Second Response to Reviewers.docx [file pone.0292695.s004.docx]

Dear Dr Grahn,

Thank you and the reviewers for generously dedicating your time to carefully reviewing the revised manuscript and identifying further points for improvement. We have revised the manuscript in light of the feedback received, integrating all the comments provided. The subsequent text discusses each, outlining the measures we have implemented to address them. Changes made are clearly highlighted in the revised manuscript. Below, the responses are provided with comments and actions that were taken to address them. The responses are highlighted in blue.

Yours sincerely,

**Simon Hanzal, Gemma Learmonth, Gregor Thut and Monika Harvey**

Authors of PONE-D-23-30888

Probing sustained attention and fatigue across the lifespan

**Detailed Response:**

Please include the following items when submitting your revised manuscript:

- A rebuttal letter that responds to each point raised by the academic editor and reviewer(s). You should upload this letter as a separate file labeled 'Response to Reviewers'.
- Included (Second Response to Reviewers).
- A marked-up copy of your manuscript that highlights changes made to the original version. You should upload this as a separate file labeled 'Revised Manuscript with Track Changes'.
- Included (Second Revised Manuscript with Track Changes).
- An unmarked version of your revised paper without tracked changes. You should upload this as a separate file labeled 'Manuscript'.
- Included (Second Manuscript).

If you would like to make changes to your financial disclosure, please include your updated statement in your cover letter. Guidelines for resubmitting your figure files are available below the reviewer comments at the end of this letter.

Journal Requirements:

Please review your reference list to ensure that it is complete and correct. If you have cited papers that have been retracted, please include the rationale for doing so in the manuscript text, or remove these references and replace them with relevant current references. Any changes to the reference list should be mentioned in the rebuttal letter that accompanies your revised manuscript. If you need to cite a retracted article, indicate the article’s retracted status in the References list and also include a citation and full reference for the retraction notice.

Author’s response: The reference list was reviewed for retractions of papers and none were found. Slight errors in reporting the doi were corrected.

[Note: HTML markup is below. Please do not edit.]

Reviewers' comments:

Reviewer's Responses to Questions

**Comments to the Author**

1. If the authors have adequately addressed your comments raised in a previous round of review and you feel that this manuscript is now acceptable for publication, you may indicate that here to bypass the “Comments to the Author” section, enter your conflict of interest statement in the “Confidential to Editor” section, and submit your "Accept" recommendation.

Reviewer #1: (No Response)

2. Is the manuscript technically sound, and do the data support the conclusions?

The manuscript must describe a technically sound piece of scientific research with data that supports the conclusions. Experiments must have been conducted rigorously, with appropriate controls, replication, and sample sizes. The conclusions must be drawn appropriately based on the data presented.

Reviewer #1: Yes

3. Has the statistical analysis been performed appropriately and rigorously?

Reviewer #1: Yes

4. Have the authors made all data underlying the findings in their manuscript fully available?

The [PLOS Data policy](http://www.plosone.org/static/policies.action#sharing) requires authors to make all data underlying the findings described in their manuscript fully available without restriction, with rare exception (please refer to the Data Availability Statement in the manuscript PDF file). The data should be provided as part of the manuscript or its supporting information, or deposited to a public repository. For example, in addition to summary statistics, the data points behind means, medians and variance measures should be available. If there are restrictions on publicly sharing data—e.g. participant privacy or use of data from a third party—those must be specified.

Reviewer #1: Yes

5. Is the manuscript presented in an intelligible fashion and written in standard English?

PLOS ONE does not copyedit accepted manuscripts, so the language in submitted articles must be clear, correct, and unambiguous. Any typographical or grammatical errors should be corrected at revision, so please note any specific errors here.

Reviewer #1: Yes

6. Review Comments to the Author

Please use the space provided to explain your answers to the questions above. You may also include additional comments for the author, including concerns about dual publication, research ethics, or publication ethics. (Please upload your review as an attachment if it exceeds 20,000 characters)

Reviewer #1: The authors have made substantial revisions to the paper, and I believe it is much improved. I have provided some additional comments below that the authors could further consider:

Abstract:
-There should be a reference for the quoted text in the first sentence, if it comes directly from a source.

Authors’ response: We thank the reviewer for this observation. As the journal requirement specifies that no in-text referencing should occur in the abstract, the introduction of the term state fatigue was reworded (Abstract, page 2):

*“whereas state fatigue is a short-term reaction to intense or prolonged effort.”*

-Could just say “An online version” and leave the JsPsych part out of the third sentence – as this could potentially lead to some confusion about what this is referring to without further context. It seems unnecessary to note the software used to administer the task in the abstract, from my perspective.

Authors’ response: We agree with the reviewer about the redundancy of this detail in the abstract. Reference to JsPsych was crossed out (page 2) from the text:

*“An online version.”*

Reference to JsPsych now remains only in the methods section (page 11).

Introduction:
-There could be a brief description of the way that the SART is administered when this is first mentioned.

Authors’ response: We thank the reviewer for highlighting the need to describe the administration of SART, which has now been included at the first mention of the task (Introduction, page 5):

*“The task is characterised by frequent go trials where a response is expected and rare no-go trials which tend to elicit errors arising from attentional lapses. Performance on the SART is thus typically measured in terms of response accuracy with a focus on commission errors (i.e., when erroneous responses are made in no-go trials), reaction times, and standard deviations in reaction times.”*

-The conceptual distinction between energy and fatigue could still be further described in the introduction.

Authors’ response: We thank the reviewer for noting this need. More text was added in order to better outline the difference between the two concepts and define energy (Introduction, page 4):

*“Although the two scales seem to be closely related, findings of divergent changes in both suggest that they constitute two related, unipolar aspects [13,26]. In relation to fatigue, energy was described as the potential to carry out mental or physical activity [26]. The two measures can often share a similar pattern of response to fatiguing tasks [25], but have also been found to differ, especially in the context of physical activity [26,27].”*

-The “Online Research” section feels a bit out of place. I wonder if this could possibly be combined with another section in a way that makes it less abrupt when reading the introduction. Just something to consider.

Authors’ response: We thank the reviewer for the valuable suggestion. The section was sightly shortened and integrated with the aims section (Introduction, page 6):

*“We leveraged the online approach in the present investigation and hypothesised that in an online experiment, trait fatigue could either negatively affect task performance directly, or that trait fatigue would predispose participants to higher levels of pre-task state fatigue.”*

-suggested edit for the aims: “Specifically, we predict that no-go accuracy…”

Authors’ response: We thank the reviewer for the observation. The text was changed to match the suggested edit (Introduction, page 6).

Methods
-What is the relevance of the statement: “All European participants completed the study in the morning…” Why was this done? Also, did non-European participants complete the study at a different time? This is mentioned again later, but I am wondering why there is a distinction in here on the European vs. non-European participants.

Authors’ response: We thank the reviewer for raising this key point. The original text was referring to participants accessing the study from time zones British Summer Time (BST) and Central European Summer Time (CEST), covering most of the land area of Europe. Since the study was released at 8am BST and completed within two hours, it meant that majority of participants completed the study in the same time period of the day, although the study was released globally. This fact is important as both fatigue and energy may be affected by circadian rhythm (Lerdal et al., 2013). The text was extended to explain the reasoning behind this detail and a distinction was made to specify that “European” in this context refers to time zones (Methods, page 15):

*“This also meant that since most of the participants who completed the study accessed it from European countries under either British Summer Time (BST) or Central European Summer Time (CEST), the predominant majority of participants completed the study in the morning.”*

Results:
-It is more conventional to include the statement on informed consent in the methods section, rather than the results.

Authors’ response: We agree with the reviewer about the correct location of the statement within the text. It has been transferred from the results (page 15) to the methods (page 10).

-In the text (e.g., page 16, line 341), a beta value of < .001 is noted for the fatigue change predictor. However, inspection of the supplementary tables shows that this is the unstandardized coefficient. There are other examples of this elsewhere in the results section. I suggest that the standardized beta coefficients can also be added to the tables, to provide further context for the size of the effects regardless of measurement tool scale in the regression analyses. Given the very small values for the unstandardized coefficients, I think it could be helpful to also include the standardized coefficients.

Authors’ response: We thank the reviewer for pointing out the importance of including fuller information on the re-run models in the supplementary tables. We agree that the small values, although not incorrect, are not very informative. Therefore, the standardized coefficients were obtained from the original script using the R Package lm.beta (Behrendt, 2023) and added into a new column in all tables of the appendix (S1 Appendix). Unstandardized coefficients were supplanted by standardized coefficients in the text where applicable. Furthermore, negative and positive values of beta coefficients and t-values in the appendix tables were aligned with those in the text:

page 17: *“The overall model was significant F(10, 104) = 2.51 p = .010, R2 = .190 and only fatigue change was found to be a predictor of accuracy change with a negative relationship between accuracy change and fatigue change, β = -.359, t = -2.82, p = .006,”*

page 18: *“Only age was found to be a predictor of accuracy, β = .372, t = 3.64, p < .001:”*

page 18: “*only block number found to be a predictor of reaction time, β = -.385, t = -8.94, p < .001*,”

page 20: *“Pre-task state fatigue was positively related to mental fatigue (β = .187, t = 2.26, p = .026), physical fatigue (β = .351, t = 3.05, p = .003) and general fatigue (β = .731, t = 6.75, p < .001), but was not related to reduced activity (β = .116, t = 1.10, p = .275) or reduced motivation (β = .088, t = .880, p = .380).”*

page 20: “*Pre-task state energy was negatively related to general fatigue (β = -.672, t = -6.47, p < .001) and reduced activity (β = -.371, t = -3.66, p < .001), whilst showing no relationship to mental fatigue (β = -.090, t = -1.14, p = .256), physical fatigue (β = .211, t = 1.90, p = .060) or reduced motivation (β = .096, t = 1.01, p = .317).*”

pages 20-21: “*State fatigue change was negatively related to reduced activity (β = -.340, t = -2.31, p = .023), with a small effect size. This meant that participants who had higher reduced activity scores had a smaller increase in their state fatigue during the task. There were no other relationships between state fatigue change and any of the other 4 MFI subscales: mental fatigue (β = -.109, t = -.950, p = .344), physical fatigue (β = -.129, t = -.803, p = .424), general fatigue (β = .098, t = .648, p = .518) or reduced motivation (β = .250, t = 1.80, p = .074). The same model was run to predict state energy change from the five MFI subscales (F(5, 109) = 3.10 p = .012, R2 = .125). Again, reduced activity was also a positive predictor of energy change, β = .377, t = 2.60, p = 0.01 with a small effect size. This showed that participants with higher reduced activity scores reported less energy loss during the task. No relationship was found with the other scales: mental fatigue (β = .046, t = .403, p = .688), physical fatigue (β = .131, t = .829, p = .409), general fatigue (β = -.051, t = -.341, p = .734) and reduced motivation (β = .212, t = 1.55, p = .124).”*

Since a new statistical package was utilised to compute the standardised beta coefficients, a reference to it has been added in the relevant part of the results section:

page 12: “‘*moments’ [65] and ‘lm.beta’ [66].”*

During the process of updating the tables, we noticed minor rounding and transcription inconsistencies when comparing the output of the script to the submitted tables. Therefore, we updated the tables to have the correct readings from the output of the models. It should be noted that these corrections bear no impact on the results of the analysis as reported in the paper.

Discussion:
-I wonder about another potential explanation for the finding that older participants had better nogo performance than younger participants; and that greater fatigue change was associated with poorer nogo performance. Does this mean also that younger people had greater fatigue change with time on task? That is perhaps also somewhat unexpected.

Authors’ response: We thank the reviewer for the valuable reflections about the effects. Answering the first question, it is interesting to note that age failed to be a significant predictor of no-go accuracy change (β = .086, t = .860, p = .391 in S1 Appendix). While we agree with the editor that the findings would point to this added effect, its absence likely arose due to the number of factors included in the various linear models. Since the section in the results already specified which factor affected no-go accuracy change, it would be repetitive to list these again in the discussion.

Another potential factor that should be considered in the discussion is how the at-home, online nature of the task may have resulted in these findings. The use of the remote delivery of the test is discussed, but primarily in the context of its usefulness for engaging in research that uses an online delivery method. However, this approach could also have a different impact on fatigue and performance in younger vs. older individuals, possibly due to novelty of the experience. Perhaps, younger individuals, who may potentially engage in online activities more frequently and proficiently in some regard, may also engage in multitasking more frequently when engaging in analogous activities. Therefore, maintaining attention towards a single, relatively simple task of this type may lead to greater distractibility and be more mentally draining for younger individuals (maybe they were even multitasking a bit – was this possible or monitored?). In contrast, older individuals may find this experience to be more novel (and maybe more “formal”), and therefore may not be as distractable or find the task as mentally draining to engage with for a prolonged period of time. This speculation leads me to wonder if the results would be replicated in the in-lab setting. What contexts might change these findings? It could be worth further considering this and adding to the discussion along these lines.

Authors’ response: We agree that literature aligns in its discussion of the potentially confounding effect of a familiar environment on age differences (Ryan & Campbell, 2021). These are primarily detected in the context of in-person laboratory studies and do contrast with participants sampled exclusively from Prolific, a platform relying on stable samples committed to long-term engagement with online research. We see the reality of this difference may manifest in future attempts to generalise the findings from the online environment to in-person lab confirmations, where the older sample may have an underlying age difference. The warning has been added to text (Discussion, page 26):

*“Furthermore, older participants in the present sample may have different motivational levels, an effect previously seen in laboratory samples [80]. Future replications should aim to survey motives for participation and rates of mind-wandering to isolate potential age-related confounds.”*

Distractibility was not subjectively measured in the present investigation. As an objective measure, the methods do specify conditions when a task block would be considered invalid due to multitasking, or when the participant would click away from the survey (page 13). A further examination of total survey duration time revealed no relationship between fatigue change scores and survey duration (F(1, 113) = 3.60, p = .060) and neither was there any relationship between energy change scores and survey duration (F(1, 113) = .01, p = .923). This suggests that while all participants have utilised the breaks available throughout the survey and between the blocks, the extended time to take these breaks likely did not affect the experiment beyond what is typically displayed in equivalent laboratory settings. The need to track distractibility, or mind-wandering in future investigations was again addressed in the discussion (page 26):

*“Future replications should aim to survey motives for participation and rates of mind-wandering to isolate potential age-related confounds.”*
